# Glucose metabolism and AD: evidence for a potential diabetes type 3

**DOI:** 10.1186/s13195-022-00996-8

**Published:** 2022-04-20

**Authors:** Andrea González, Camila Calfío, Macarena Churruca, Ricardo B. Maccioni

**Affiliations:** 1 Laboratory of Neurosciences and Functional Medicine, International Center for Biomedicine (ICC), Avda. Vitacura 3568, D 511-512, Vitacura, Santiago Chile; 2 Faculty of Sciences, University of Chile, Las Encinas 3370, Ñuñoa, Santiago Chile; 3 Department of Neurology, Faculty of Medicine East Campus Hospital Salvador, University of Chile, Salvador 486, Providencia, Santiago Chile

**Keywords:** Glucose metabolism impairment, Tau posttranslational modifications, Insulin resistance, ER stress, Alzheimer’s disease

## Abstract

**Background:**

Alzheimer’s disease is the most prevalent cause of dementia in the elderly. Neuronal death and synaptic dysfunctions are considered the main hallmarks of this disease. The latter could be directly associated to an impaired metabolism. In particular, glucose metabolism impairment has demonstrated to be a key regulatory element in the onset and progression of AD, which is why nowadays AD is considered the type 3 diabetes.

**Methods:**

We provide a thread regarding the influence of glucose metabolism in AD from three different perspectives: (i) as a regulator of the energy source, (ii) through several metabolic alterations, such as insulin resistance, that modify peripheral signaling pathways that influence activation of the immune system (e.g., insulin resistance, diabetes, etc.), and (iii) as modulators of various key post-translational modifications for protein aggregation, for example, influence on tau hyperphosphorylation and other important modifications, which determine its self-aggregating behavior and hence Alzheimer’s pathogenesis.

**Conclusions:**

In this revision, we observed a 3 edge-action in which glucose metabolism impairment is acting in the progression of AD: as blockade of energy source (e.g., mitochondrial dysfunction), through metabolic dysregulation and post-translational modifications in key proteins, such as tau. Therefore, the latter would sustain the current hypothesis that AD is, in fact, the novel diabetes type 3.

## Introduction

Alzheimer’s disease (AD) is the most common dementia with 60–70% of cases. Behavioral changes and cognitive impairment resulting from neurodegeneration are observed, possibly 10 years after the onset of proteinopathy. The two characteristic phenomena, used as biomarkers [1] are the presence of Ab plaques and hyperphosphorylation of tau that self-aggregates, forming neurofibrillary tangles (NFT) [2, 3]. However, AD is a multifactorial disease with a complex etiology. Two major groups of patients are evidenced: early-onset or familial with a hereditary component due to genetic mutations that alter the amyloid precursor protein (APP) or presenilins 1 and 2. The second group, late-onset or sporadic AD, occurs in 97% of cases. It is associated with multiple factors such as the polymorphism of apolipoprotein E (APOE) gene, presenting the APOε4 allele, hyperlipidemia, hypertension, type II diabetes, and coronary disease [4]. Age is one of the main associated risk factors for developing sporadic AD [5]. Different molecular events are involved. One of them is the misfolding of proteins due to the stress of the endoplasmic reticulum and failures in its quality control system of response to unfolded proteins (UPR) [6]. This enables for the accumulation of NFT.

Another alteration associated with age, due to the fact that neurons have a decreased capacity to regenerate, is the decrease in the amount of available energy [7]. This is due to two main reasons: the glucose transporters (GLUT) are expressed in less quantity [8] and alterations in insulin signaling [9].

The human brain uses 20% of the glucose [10]; it needs a lot of energy principally to support the synaptic activity [11]; 95% of glucose is used in the production of ATP [12]. This is why alterations in glucose metabolism cause damage to cell regulation, as decreased ATP can affect proper synaptic function [4]. Much of the process is independent of insulin regulation. However, there are insulin receptors in various brain areas, influencing processes such as memory, cognition, and regulation of energy metabolism [13, 14]. Insulin resistance significantly increases the risk of developing sporadic AD [15, 16], while type II diabetes (DTII) increases the risk of AD by 50% [17].

In addition, it has been shown that the development of insulin resistance and DTII may be mediated by endoplasmic reticulum (ER) stress by activating c-Jun N-terminal kinases (JNKs), and this in turn triggers downstream signaling cascade activity [18] of the inflammatory type [19], due to the excessively prolonged response to unfolded proteins (UPRs) by the stressed ER [19]. It has been shown that epigenetic variations such as glycosylations disturb protein folding and trigger ER stress [19]. All the latter also influence post-translational modifications in tau that propitiate tau self-assembly, from an a-helix to a b-sheet structure [20]. This b-sheet conformation is the one prone to form aggregates [20]. Increased phosphorylation, decreased ubiquitination, and decreased methylation are some of the post-translational changes observed in AD [21–23]. Remarkably, lysine methylation is decreased in AD patients [22], which may be explained by the decreased glucose uptake due to the downregulation of the GLUT receptors [24]. The latter is correlated with tau hyperphosphorylation [24], leading to the final outcome of neurodegeneration and neuroinflammation.

Here, we review how alterations in glucose metabolism stress the endoplasmic reticulum and this, in turn, influences the post-translational modifications of the tau protein associated with AD.

## Metabolic alterations in the pathogenesis of AD

### Endoplasmic reticulum (ER) stress

The cell has intracellular organelles that fulfill different functions, for example, in the nucleus the transcription of DNA to RNA [25]. In the ER, proteins are synthesized and subsequently transported to the Golgi apparatus, before their subsequent destination [26]. The ER has an intracellular membrane system, with a unique quality control system that allows the management of protein aggregates [27] and whose purpose is to maintain proteostasis [28].

ER stress is understood as the imbalance between ER protein folding capacity and the demand for protein synthesis, resulting in the accumulation of misfolded proteins in the ER lumen (misfolded) [29–31]. This can be produced by pathological conditions that can disrupt ER function, such as changes in the availability of Ca^+2^, ATP, or pathogens that release of misfolded or unfolded proteins (UPs) [32]. In the latter case, misfolded proteins or UP are directed towards a degradation pathway present in the ER called ERAD (ER-associated degradation) [33, 34]. UPs are recognized by chaperone proteins, which together with other proteins, stabilize unstable forms [35].

The ER is stressed when the amount of misfolded proteins exceeds its containment capacity, so mechanisms are displayed to correct this error [36]. A cellular UP response program (UPR) is activated to manage this error that acts through the reduction of the synthesis of new proteins and the transcription of chaperones and activates the degradation of UP in the proteasomes [27, 29, 37, 38].

Due to ER stress, sensors of the UP response are activated and decrease protein translation: IRE1 (endoribonuclease that requires inositol), PERK (protein kinase RNA-like endoplasmic reticulum kinase), and ATF6 (that activates transcription factor 6) [27, 32, 37, 39]. Then, the chaperone binding immunoglobulin protein (BiP) binds and inhibits one of the aforementioned transduction proteins, binding to misfolded proteins and adaptation to UP overload [32]. In this case, the UPR is not able to reestablish the folding equilibrium, and ER stress eventually leads to apoptosis [27, 40].

When the UPR is surpassed due to the excessive accumulation of misfolded proteins derived from environmental factors such as aging [31, 41], and genetic mutations, it could lead to diseases such as diabetes type 2 (DTII), atherosclerosis, and neurodegenerative diseases, e.g., AD, which is associated with abnormal protein folding [42].

Misfolded proteins that aggregate intracellularly constitute a hallmark in the pathogenesis of neurodegenerative diseases. It is likely that the ER regulatory mechanisms are bypassed, allowing self-aggregation and intracellular damage [41]. A tauopathy modeling was performed in a C-elegans; AD is one of them. The vital importance of IRE1 and ATF6, two of the UPR pathways to regulate proteostasis in ER, was highlighted [41]. Furthermore, a chronic UPR response can induce apoptosis through an intrinsic mitochondrial pathway dependent on members of the pro-apoptotic B cell lymphoma (Bcl-2) family [43]. If ER stress is prolonged, it causes an alteration in insulin synthesis, while apoptosis of B cell of the pancreas has been observed in the late stages of hyperglycemia and insulin resistance [44].

The large biosynthetic load on the ER for insulin production in response to glucose (from food intake) can exceed ER folding capacity, resulting in ER stress. This leads to the consequent activation of PERK, which reduces the ER protein load by phosphorylating eIF2 (elongation initiation factor-2), a protein necessary for protein translation [44]. It has been observed that in PERK −/− cells, protein synthesis does not respond to stress, leading to the accumulation of folded proteins (for example, proinsulin) and subsequently to apoptosis. In this case, ER stress-induced apoptosis can increase inflammatory signaling. PERK −/− mice are more prone to DTII and progressive hyperglycemia [45].

ER stress has also been related to diseases promoted by misfolded proteins or proteinopathies such as Alzheimer’s. There is an accumulation of misfolded proteins exceeding the response capacity of UPR [31]; greater markers of UPR activation have been demonstrated in the post-mortem brain of subjects with AD [38, 46]. It has been shown in transgenic animal models of AD that inhibition of PERK activity in hippocampal slices facilitates mGluR-LTD and, in turn, deletion of PERK deactivates the eIF2a pathway, which has been associated with improvements in memory [47]. UPR plays a fundamental role in the neurotoxicity manifested in AD [36]. Strong evidence shows that ER stress activates signaling pathways that influence tau phosphorylation, the amyloid cascade, and synaptic dysfunction [31].

### Insulin resistance

Scientific evidence has proven that insulin signaling and glucose metabolism are altered in AD. For this reason, some authors such as Kroner [48] have designated AD as type III diabetes [16, 48].

Insulin is a polypeptide hormone made up of two chains of 51 long amino acids [49]; it is synthesized in B cells of the pancreas and regulates glucose metabolism [50], although it is also released locally in the CNS in minimal quantities [51]. It is synthesized as a prohormone, begins as a pre-proinsulin, and is eliminated in the rough ER cistern as proinsulin, and then it is directed to the Golgi apparatus, and it is packaged in secretory vesicles [52]. The proinsulin molecule dissociates as C peptide, by the enzymatic action of endopeptidases and carboxypeptidases, leaving the amino terminal peptide B linked by a disulfide bridge to the amino terminal peptide A. Then, its native structure is folded, and its conformation of two A chains and B is stabilized by the double disulfide bond [52, 53].

The peripheral areas of the body require insulin to activate the signaling that allows the translocation of glucose transporters, entering the glucose into the cell [54]. When insulin binds to the insulin receptor substrate or signaling adapter protein (IRS), it is recruited and phosphorylated [55]. The IRS activates downstream signaling pathways, of the IRS family; there are two fundamental ones: IRS1 and IRS 2. These activate two main signaling cascades such as the phosphatidylinositol 3-kinase (PI3K)-AKT/protein kinase B (PKB) pathway and mitogen-activated protein kinase (MAPK) pathways [54]. Furthermore, the expression of the activation of the PIK3 pathway together with glycosylation kinase (GSK-3) follows an expression similar to the insulin pathway in peripheral tissues [56]. The PIK3 pathway is considered an integrating pathway for insulin, and it is hypothesized that it is associated with learning and memory [50]. It was found in a group of diabetic women that they have greater cognitive impairment than women without diabetes [57]. Alterations in insulin signaling are associated with cognitive impairment [48].

In the CNS, insulin must cross the blood-brain barrier (BBB) and bind to its receptor, whose conformational change leads to the enzymatic activity of tyrosine kinase and the autophosphorylation of the receptor [58]. In the CNS, there are receptors in septum, amygdala, hypothalamus, hippocampus, cerebral cortex, and olfactory bulb [14, 59–62]. The function in the hypothalamus of the insulin receptor is through signaling of food intake and energy regulation, influencing peripheral metabolism [63]. Insulin regulates neuronal development, modulates neurotransmitter signaling pathways, and participates in learning and memory [64, 65]. Regarding memory, insulin activates signaling cascades in the hippocampus that affect synaptic plasticity [66]. Insulin resistance in the CNS has recently been found to cause anxious states, hyperphagia, and depressive-like behaviors [67]. Insulin resistance can be evaluated through the ratio between the serine-phosphorylated insulin receptor substrate with respect to the total phosphorylated insulin receptor substrate, in the brain or peripheral tissues. A greater ratio indicates increased insulin resistance [9, 68]. This marker, together with ex vivo stimulation of brain tissue with insulin, was employed to demonstrate brain insulin resistance in AD patients [9, 69].

Peripheral insulin resistance (hyperinsulinemia) decreases glucose sensitivity in major target organs such as muscles, liver, and adipose tissue. In this context, there is an increase in the amount of insulin available in the bloodstream, which consequently increases tolerance to glucose [70]. Several findings suggest that the development of these alterations would be associated with mitochondrial dysfunction and/or ER stress due to aging [71, 72].

Hyperinsulinemia is a risk factor for the development of hyperglycemia and type II diabetes (DTII). It is associated with a higher risks of neurodegeneration [15, 50, 73–75], due to a decreased degradation of amyloid beta, because the augment in the insulin sequesters by the insulin degrading enzyme (IDE).

It is also associated with an increase in CDK5 activity and, with it, the hyperphosphorylation of tau that is involved in AD [15].

Currently, presenting AD is considered to have a higher risk of DTII [76] and vice versa [36]. It is very possible to go from mild cognitive impairment to AD if glucose metabolism is altered [58, 77]. This has been evidenced as the disease progresses [78–80]. Other studies suggest an association between abnormal tau phosphorylation and insulin resistance [81]. Both diseases are related to changes in the expression of glucose transporters and in particular AD with a decrease in available energy in neurons [82]. However, the latter is still controversial, as other studies showed no relation between diabetes TII and the formation of neurofibrillary tangles (NFT) and Ab peptide in diabetic postmortem brains [83]. Nevertheless, it should be considered that this study relates to the APOE genotype and not the sporadic AD. Thus, it is possible that although glucose metabolism impairment can relate to neurodegenerative diseases, the mechanism is still inconclusive.

In the CNS, glucose bioavailability is limited by crossing the blood-brain barrier (BBB), mediated by glucose transporters GLUT1-6 and GLUT-8 and sodium-dependent transporters (SGLT1) to reach neurons and glia [82, 84–86]. Another energy source is lactate derived from astrocytes [87–89] and brain ketones [90].

In regard to the glucose transporters, the GLUT1 transporter is expressed in the endothelial cells of the BBB [91, 92]; GLUT3, on the other hand, is expressed in neurons with high affinity to glucose [93, 94], and GLUT4 is expressed in the BBB of the ventromedial hypothalamus [95] and temporal cortex; therefore, it participates in memory and cognition processes [96]. Both GLUT1 and GLUT3 are insulin independent for membrane translocation [97]. GLUT3 and GLUT4 transporters decrease their expression with aging [98].

Impairments in glucose metabolism have been reported to cause memory impairment and hippocampal atrophy [99]. However, the exact molecular mechanisms that associate the origin of AD disease with glucose and insulin metabolic alterations are still unclear [36].

Several avenues regarding treatments for insulin resistance and TII diabetes have shown an effect on AD, for example, receptor agonists for incretin (IRA) such as semaglutide, which is shown not to cross the BBB. However, this compound is still one of the most promising single IRA in the treatment of AD and Parkinson’s disease, since it is one of the most stable IRA [100]. Metformin, on the other hand, is a biguanide used as an oral antidiabetic drug. In AD, it has been demonstrated that it can act as an activator of chaperone-mediated autophagy in a mouse AD model [101]. It should be noted that autophagy is a key process in neurodegenerative diseases, as it is considered a hunter of aggregates [102]. The latter opens new therapeutic approaches that seek to induce autophagy in neurodegenerative diseases [103]. Intranasal insulin is a novel treatment for TII diabetes, which has demonstrated promising results in AD, as it improves brain insulin signaling and, consequently, ameliorates the cognitive performance and metabolic integrity of the brain in patients with AD [104].

All the latter are consistent with an association between glucose metabolism impairment and AD. Furthermore, use of fluoro (F18)-2-deoxy-d-glucose (FDG)-PET, which involves a glucose analog to evaluate carbohydrate metabolism in the brain, has been proposed as a potential biomarker [105].

## Tau post-translational modifications

### Microtubule-associate protein tau

Tau protein is a microtubule-binding protein described as a MAP that binds tubulin [106]. Due to the plasticity of its encoding gene (Chr. 17, region 17q 21 in humans), some of its exons (2, 3, 4A, 6, 8, 10, and 14) can be processed by alternative splicing, thus generating several isoforms [107]. In humans, 6 isoforms have been described, which consists of two domains, an amino terminal domain and a carboxy-terminal domain [108]. The first one is denominated “projection domain,” which is rich in proline and it also has an acid region. The C-terminal is the principal binding domain of tau, and it contains three (3R) or four (4R) internal repeats [108].

In the central nervous system, tau under normal conditions provides stability to the microtubules (MT) and articulates the transport system of signaling molecules and cellular components [109]. Those functions are disrupted during the course of AD, due to several changes in the pattern of post-translational modifications.

### Post-translational modifications

Tau protein can suffer phosphorylations, methylations, ubiquitinations, and glycosylation/truncation, post-translational modifications that generate different tau variants.

#### Phosphorylation

Tau has over 30 aa that can be phosphorylated, which includes ser, thr, and tyr [110]. In AD, tau is hyperphosphorylated, which captures native tau and other microtubules-associated proteins, causing the disassembly of microtubules [110].

Subsequently, there is a destabilization of the cytoskeleton, produced by the alteration of tau-dependent cellular functions, such as vesicular and organelle transport, axonal growth, and nerve signal propagation. This anomaly is known as tauopathies and is present in many neurodegenerative diseases [111]. There are 20 diseases categorized as tauopathies, which are sub-divided into two groups, a primary and a secondary; Alzheimer disease is part of the secondary group, and it is also the most preponderant [111]. The secondary group is characterized for the presence of both intracellular tau pathology and extracellular amyloid plaque deposits [111]. The particularity of this tauopathy is the formation of insoluble deposits called neurofibrillary tangles (NFT) in the three (3R) and four (4R) isoforms [112]. The dysfunctionality caused by NFT is manifested from the soma to the dendrites, and the most commonly affected regions of the brain are the entorhinal cortex, the hippocampus, and the neocortex [112].

Studies have demonstrated how an increased activity of kinases, such as CDK5, and downregulation of phosphatases influences tau hyperphosphorylation, leading to the oligomer formation of tau [113, 114]. The deregulation of CDK5 is due to the formation of CDK5/p25 complex, product of p35 splitting, possibly as a result of oxidative stress and amyloid peptides to which the neuron has been exposed [115, 116]. The latter leads to the proteolysis of p35, transforming into p25, a fragment of the protein that is neurotoxic and has an active and a totally extended conformation [115]. The conformational change that took place and the generation of p25 impacts the way CDK5 activates, given the fact that the latter activation lasts longer than p35 [115]. This conversion results in CDK5 hyperactivity and subsequently, a possible hyperphosphorylation of tau protein and neurofilaments, along with a cytoskeletal alteration and eventually neuronal death [117].

Their conformational structure changes from an a-helix to a b-sheet structure, which facilitates the formation of the oligomers [20].

The latter is the basis of the neuroimmunomodulation theory, proposed by our laboratory [118–120]. Indeed, fragments from paired-helical filaments (PHF) (with hyperphosphorylated tau) and other molecules (such as Ab peptides and advanced glycation end products (AGEs)) may act as a “danger signal,” activating the resting microglia [120, 121]. Activated microglia increases the pro-inflammatory signaling through the NFkb pathway, leading to the increased activation of several kinases, such as CDK5 and GSK3b [122]. The latter increases tau phosphorylation, in a cyclic course of events that eventually leads to chronic neuroinflammation and neurodegeneration [122].

It should be noted, however, that not only the hyperphosphorylation is pivotal on AD, but also which of the putative phosphorylation sites are target of the kinases. Furthermore, it was demonstrated in vitro that oxidative stress promotes tau dephosphorylation at the Tau1 epitope in SHSY5Y cells [123]. The latter was dependent on the activity of the cdk5/p35 complex, since an increase in the substrate phosphorylation as well as for the complex association was observed [123]. Also, oxidative stress induced a decrease in the amount of inhibitor-2 bound to phosphatase PP1, associated to an increased phosphorylation of the inhibitor-2 protein.

Thus, hyperphosphorylation of tau relies in a shift of balance between the kinases and phosphatases, in which the upregulation of the kinases activity exceeds the phosphatase activity.

#### Methylation

Methylation is the enzymatic addition of methyl (CH3) groups to protein substrates [124]. In this case, methyltransferases transfers the methyl group from the s-adenosyl methionine to the target residues: lysine or arginine [124]. In tau protein, this post-translational modification can play different roles during the pathological processes leading to AD [22]. It has been described lysine methylation is an endogenous post-translational modification that modulates tau aggregation [22]. In vitro studies showed that Lys methylation impaired total filament length in a stoichiometry-dependent manner [22]. Moreover, mono-methylation and di-methylation of tau are related to normal aging and AD, respectively [22]. It should be noted that several lys are next to ser/thr, which are the key aa involved in phosphorylation.

In a mass spectrometry analysis of PHFs derived from AD brains, several lysine residues were detected distributed in the projection domain and the microtubule-binding domain (MBD), which are susceptible to be methylated [125]. Also, aggregated tau derived from AD brains is monomethylated at seven lysine residues in the proline-rich region and the R1/R2 repeats of the MBD [125]. Of these residues, the most frequently methylated ones are K180 and K267, in contrast to K290 which is has the lowest level of methylation [125]. Interestingly, in PHF-tau, phosphorylation of S262, which reduces tau affinity for microtubules, is found more frequently in the presence of methylated K267 [125].

Also, in PHF-tau, another residue, K254, was found to be mainly methylated and, in a lesser extent, ubiquitylated [125]. The latter suggests that methylation may prevent tau degradation by the proteasome.

All the latter suggests that tau lysine mono-methylation leads to a confirmation that allows self-assembly and aggregation. Considering lysine methylation is an apolar post-translational modification, it could be possible that the shift in the patter of methylation, from di-methylated to mono-methylated, exposes the residues susceptible for phosphorylation, blocking the residues for ubiquitination and, consequently, will be susceptible for self-assembly.

#### Ubiquitination

The quality control of the proteins realized by the ubiquitin-proteasome system (UPS) is fundamental. Ubiquitination is the specific binding of ubiquitin, a small 8.6 kDa regulatory protein to tag proteins for degradation by the UPS. Proteins that will be eliminated are poly-ubiquitinated and identified by the proteasome for their degradation [126].

As several other neurodegenerative diseases, a major trademark of AD is the accumulation of misfolded proteins. In non-pathological conditions, tau is ubiquitinated and processed in the proteasome [127]. In AD, it has been demonstrated that the ubiquitin-proteasome pathway is impaired and dysfunctional [128]. Since the ubiquitin-proteasome system is pivotal in tau degradation, its impairment leads, consequently, to tau accumulation [129]. It should be noted that the first step in the ubiquitin-proteasome system is the activation of ubiquitin in an ATP-dependent manner, mediated by the ubiquitin-activating enzyme (E1) [130]. Thus, if less intracellular ATP is generated due to the mitochondrial dysfunction, less ubiquitination will occur. This would explain, at least in part, the accumulation of aggregated tau proteins.

#### Glycosylation/truncation

These modifications include glycosylation and truncation, both of which occur in early stages of AD.

In regard to glycosylation, this post-translational modification is the covalent attachment of oligosaccharides to a protein, tau in this case. Glycosylation of tau protein was non-physiological in the brain of AD patients, and this abnormal pattern of glycosylation was not detected in control patients [131]. In other study, Liu et al. [132] have shown that abnormal in vitro glycosylation modulates the phosphorylation of tau by the kinases PKA, GSK-3, and CDK-5, which, in turn, inhibits dephosphorylation by the phosphatases PP2A and PP5 [133]. The latter is closely related to the negative correlation between O-glycosylation of tau and its phosphorylation [132, 134]; thus, interaction between many post-translational modifications may be necessary to induce the oligomer tau formation.

Truncation is another post-translational modification that enhance the capacity of tau to aggregate [135]. In AD patients’ brains, this process occurs in D13, E391, and D421 [136]. The latter leads to an accumulation of tau protein truncated at D13, E391, and D421, which correlates with AD progression [136]. These truncated tau forms are found in PHFs [137], and tau cleavage occurs after its hyperphosphorylation [138]. Indeed, an in vitro model of ethanol-induced neuronal apoptosis, tau hyperphosphorylation, occurs before its cleavage, and both tau hyperphosphorylation and apoptosis are blocked by lithium [138].

## Neuroinflammatory mechanisms involved in microglial activation

### Extracellular ATP role

ATP is an intracellular signaling molecule, released into the extracellular medium when there is damage to the CNS from injured cells. Extracellular ATP activates the microglia through P2X (ionotropic) purinergic receptors [139] and induces cytokine release in the microglia [140]. In the CNS, ATP is released from glial cells and nerve terminals, functioning as a neurotransmitter or intracellular signaling [139].

Microglia migrates to where the damage is by promoting tissue repair but in turn propels excess inflammatory processes and can release neurotoxic factors that can increase neurodegeneration [141].

Extracellular ATP is a key player in the control of neuronal activity through a microglia-driven negative feedback [142]. Indeed, microglia suppresses neuronal activation through its capacity to sense and catabolize extracellular ATP, which is released upon neuronal activation by astrocytes and neurons [142]. ATP is catabolized by the microglial hydrolyzing enzymes CD39 and CD73 into AMP and adenosine respectively. Adenosine, then, suppresses the neuronal activity via the adenosine receptor A present in neurons, thus establishing the microglia-mediated negative feedback mechanism [142].

In a β-amyloid (Aβ1-42)-based mouse model of early AD, it has been demonstrated an increased release of ATP from neurons coupled to an increased density and activity of ecto-5′-nucleotidase (CD73)-mediated formation of adenosine selectively activating A2AR [143]. Moreover, CD73 inhibition impaired long-term potentiation (LTP) in mouse hippocampal slices [143].

All the later suggests that extracellular ATPs, and more specifically, adenosine, are danger signals that might be involved in synaptic loss. Consistent with the latter, ATP release from nerve terminals is increased after intracerebroventricular Aβ1-42 administration, together with CD73 and A2AR upregulation in hippocampal synapses. Importantly, this increased CD73 activity is critically required for Aβ1-42 to impair synaptic plasticity and memory since Aβ1-42-induced synaptic and memory deficits were eliminated in CD73-KO mice [143]. These observations establish a key regulatory role of CD73 activity over neuronal A2AR and imply CD73 as a novel target for modulation of early AD. On the other hand, a 50% decrease in ATP production has been observed in late-onset AD [79]. The latter is also linked to decreased glucose uptake and decreased ubiquitination.

It should be noted, however, that all the mechanisms mentioned above are indirect effects and further studies are required to fully stablish the role of extracellular ATP in AD. For that, it would be relevant to evaluate the concentration of adenosine by fluorescence and the activity of the receptors by electrophysiology.

## Conclusions

The influence of glucose metabolism is evident in several aspects of AD. First is through insulin resistance and diabetes with ER stress supported by medical evidence. This is also consistent with the fact that ER stress is also part of the UPR when accumulation of misfolded proteins occurs, such as Aβ peptide, and tau protein in AD. A pivotal part of this mechanism is also the post-translational modifications as they are key regulators of protein folding and degradation. In the case of tau protein, methylation and phosphorylation are the main regulators of tau self-assembly and aggregation. But other post-translational modifications, such as ubiquitination, are also relevant. The glucose metabolism is also involved in neuroinflammatory mechanisms regarding neurodegeneration and extracellular ATP, which is the source for adenosine generation. Adenosine, through its receptor in the neurons, stops neuronal activity, thus promoting neuronal decline and upregulation of apoptotic pathways.

**Fig. 1:**
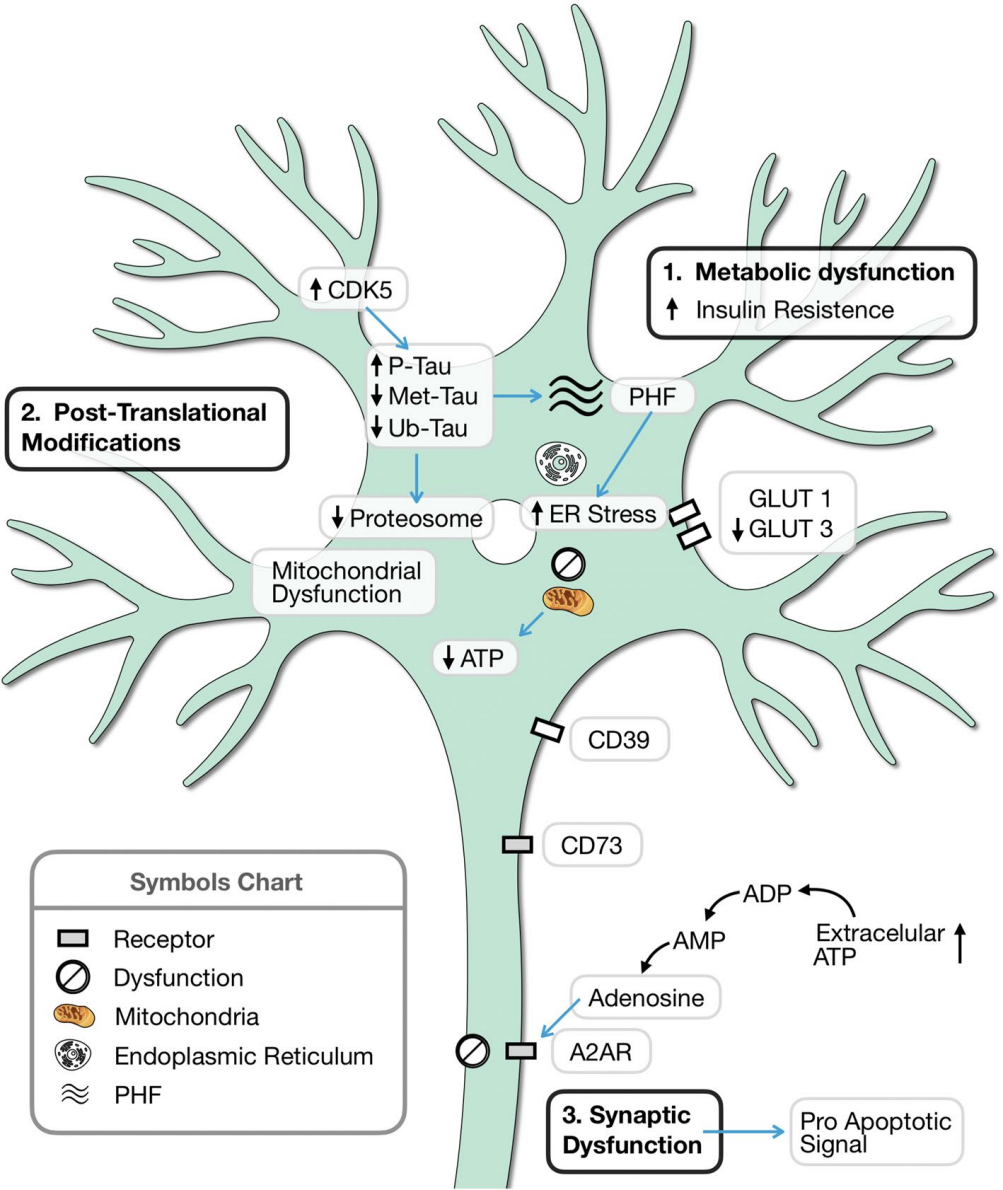
Glucose metabolism and its involvement in AD. There are several mechanisms in which, directly or indirectly, glucose is involved in AD. (i) As a potential mechanism, a diagram with a general metabolic dysfunction that leads to an increased insulin resistance, and consequently, lower glucose uptake; (ii) in post-translational modifications in which glucose or glycans are required, such as methylation. These modifications also alter others post-translational modifications, such as phosphorylation and ubiquitination, which leads to tau aggregation and (iii) through the generation of ATP, that is released to the extracellular, where it can be sensed by microglia, and then transformed into adenosine. This adenosine suppresses neuronal activity and in the long term, causes synaptic dysfunction. ER, endoplasmic reticulum; ADP, adenosine di-phosphate; A2AR, adenosine receptor type 2; GLUT, glucose transporter ; PHF, paired helical filaments; CDK5, cyclin-dependent kinase type 5; CD73: 5′-nucleotidase; CD39, ectonucleoside triphosphate diphosphohydrolase-1

Then, there are several mechanisms that overlap and fail, facilitating the aggregation of tau protein. One of them is the regulation of the glucose mechanism and the concomitant loss of insulin sensitivity. Both alter the capacity of the ER by decreasing the amount of energy available. Together with this, the mechanisms to avoid protein aggregation, driven by the chaperone proteins in the ER, are insufficient [144]. In addition, by decreasing the amount of energy, the ubiquitination that tags tau for degradation decreases. All alterations of the protein are related to dysmetabolism. Both tau hyperphosphorylation and metabolic alterations are situated in an inflammatory scenario that promotes neurodegenerative processes by activating microglia [145]. If we consider all the summarized above, during the course of AD, glucose metabolism is a key mediator that promotes a metabolic dysfunction, which lead to protein aggregation, and consequently, neuronal death is gradually reached (Fig. 1). In this review, we highlight several mechanisms involving glucose metabolism impairment that act collectively in the etiology and progression of AD, which is why it is currently accepted that AD is a novel diabetes type 3.

## Data Availability

Non-applicable.

## Abbreviations

AD: Alzheimer’s disease
NFT: Neurofibrillary tangles
APP: Amyloid precursor protein
UPR: Unfolded protein response
APO-E: Apolipoprotein E
GLUT: Glucose transporters
ATP: Adenosine-tri-phosphate
DTII: Diabetes type 2
JNKs: c-Jun N-terminal kinases
ER: Endoplasmic reticulum
UP: Unfolded proteins
ERAD: Endoplasmic reticulum associated degradation
eIF2: Elongation initiation factor-2
CNS: Central nervous system
PI3K: Phosphatidylinositol 3-kinase
MAPK: Mitogen-activated protein kinase
GSK-3: Glycosylation kinase
BBB: Blood-brain barrier
MT: Microtubules
PHF: Paired-helical filaments
MBD: Microtubule-binding domain
AGEs: Advanced glycation end products
UPS: Ubiquitin-proteasome system
LTP: Long-term potentiation
